# Advances in the Study of CircRNAs in Tumor Drug Resistance

**DOI:** 10.3389/fonc.2022.868363

**Published:** 2022-05-09

**Authors:** Song Wang, Long Qian, Tingting Cao, Li Xu, Yan Jin, Hao Hu, Qingsheng Fu, Qian Li, Ye Wang, Jiawei Wang, Yabin Xia, Xiaoxu Huang

**Affiliations:** ^1^ Department of Gastrointestinal Surgery, The First Affiliated Yijishan Hospital of Wannan Medical College, Wuhu, China; ^2^ Key Laboratory of Non-coding RNA Transformation Research of Anhui Higher Education Institution, Wannan Medical College, Wuhu, China

**Keywords:** circRNA, tumor chemotherapy, drug resistance, therapeutic target, cancer biology

## Abstract

Recent studies have revealed that circRNAs can affect tumor DNA damage and repair, apoptosis, proliferation, and invasion and influence the transport of intratumor substances by acting as miRNA sponges and transcriptional regulators and binding to proteins in a variety of ways. However, research on the role of circRNAs in cancer radiotherapy and chemoresistance is still in its early stages. Chemotherapy is a common approach to oncology treatment, but the development of tumor resistance limits the overall clinical efficacy of chemotherapy for cancer patients. The current study suggests that circRNAs have a facilitative or inhibitory effect on the development of resistance to conventional chemotherapy in a variety of tumors, suggesting that circRNAs may serve as a new direction for the study of antitumor drug resistance. In this review, we will briefly discuss the biological features of circRNAs and summarize the recent progression of the involvement of circRNAs in the development and pathogenesis of cancer chemoresistance.

## Background

**Figure 1:**
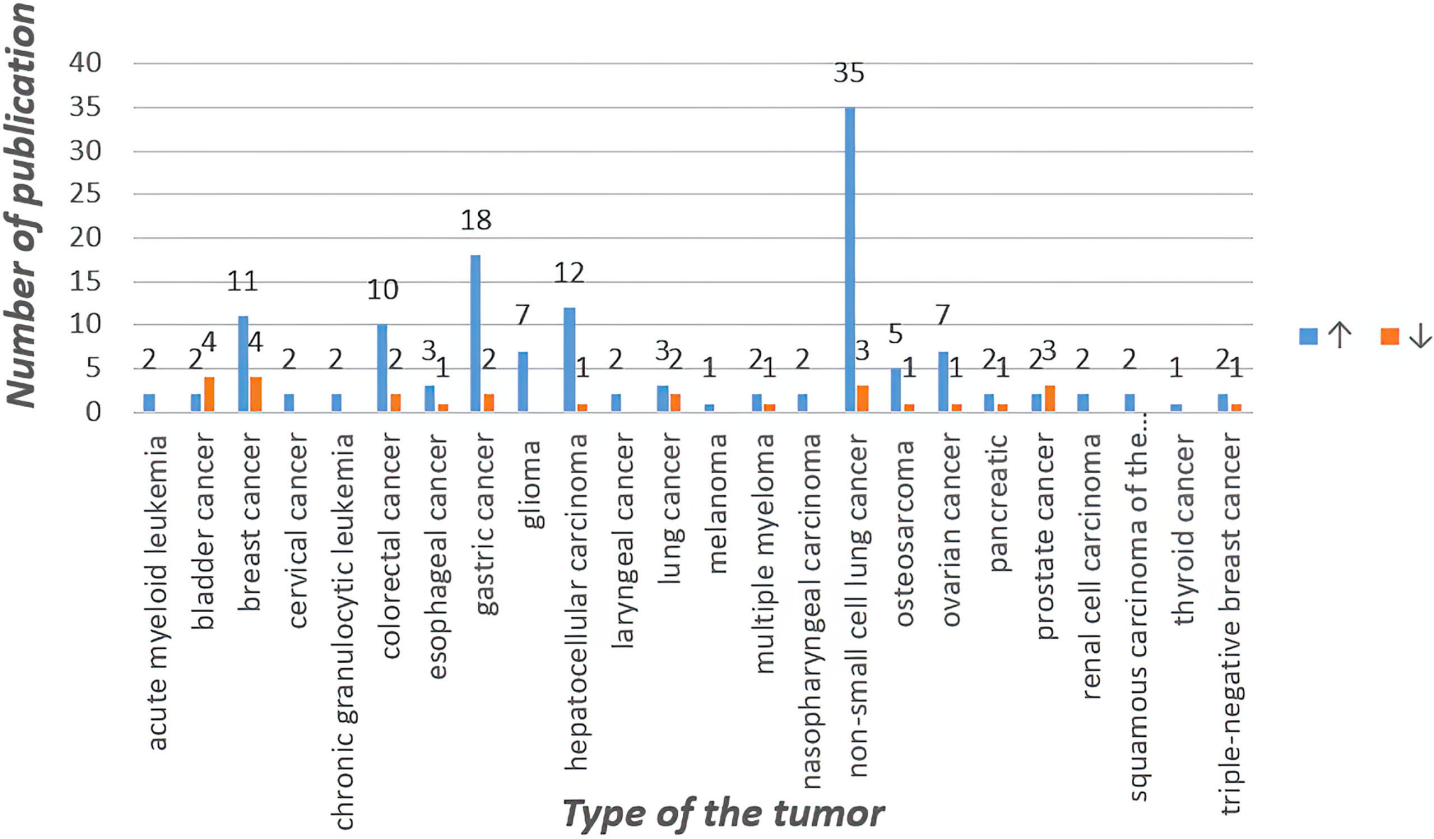
Statistics of circRNA related to chemotherapy resistance in different tumors in recent five years.

Circular RNA was first found in plant viroid (1), yeast mitochondrial RNA (2). Unlike linear RNA with 5 ‘cap and 3’ tail as terminals, circular RNA is characterized by a covalently closed loop structure with neither 5’to 3 ‘polarity nor polyadenylate tail. This inherent feature led to the widespread underestimation of circular RNA in previous polyadenylated transcriptome analysis. With the advent of specific biochemical and stoichiometric methods, a large number of circular RNA formed by reverse splicing of exons have been found in different cell lines and different species (3). Among them, circular RNA(circRNA), along with their modifiers, have been investigated to play key roles in regulating tumor development and mediating therapy resistance within various cancers, such as hepatocellular carcinoma, breast cancer, lung cancer, gastric cancer, etc. First, almost all of the reported circRNA functions through four classic mechanisms, including:1, miRNA sponge mechanisms 2, sponging of RBPs (RNA-binding proteins)3, regulation of transcription 4, translation into peptides or proteins. In addition, two limitations regarding the generation of tumor drug resistance are raised. First, the existing reports on circRNAs in cancer chemoresistance are limited to a few classical drugs and a few types of cancers. Second, most reported circRNAs, limited to those identified in cancer, remain unstudied in the context of the cancer therapeutic response, particularly radioresistance, and numerous unidentified circRNAs remain to be studied. As these limitations are gradually overcome, potential interventions targeting circRNAs are expected to overcome cancer resistance to radiation and chemotherapy (4, 5). We searched for “circRNA”, “cancer” and “drug resistance” in Pubmed, and obtained a total of 351 relevant papers, and then identified the papers that fit the topic of this paper by preliminary screening of these 351 papers, and the results were 158. According to recent studies, circRNAs mainly (1) upregulate drug efflux transporters or downregulate influx channels, (2) enhance epithelial-mesenchymal transition (EMT) and stemness, (3) alter the copy number of target genes and activation of bypass pathways or downstream signaling, (4) alter endoplasmic reticulum stress, autophagy and phagocytosis, (5) enhance or inhibit DNA repair, (6) impact the tumor microenvironment. Other factors may induce tumor drug resistance but will not be discussed here (4). In this paper, we review the possible tumor drug resistance mechanisms associated with circRNAs and their research progress in the context of chemoresistance by categorizing and summarizing a large number of relevant studies. It also discussed the limitations of available knowledge and future potential directions. The statistics are shown in **Figure 1** and **Table 1**.

**Table 1:** CircRNAs in tumor drug resistance.

Name of the tumor	CircRNA	Target(s)	Drugs	Alteration	References
nasopharyngeal carcinoma	hsa_circ_0002346	miR-422a, FOXQ1 protein	Docetaxel	↑	(6)
	hsa_circ_0004771	miR-515-5p, IL-25	DDP	↑	(7)
colorectal cancer	hsa_circ_0005963	miR-122, pyruvate kinase M2 isoform	OXA	↑	(8)
	hsa_circ_0001451	miR-18b-5p	OXA	↓	(9)
	hsa_circ_0084443	miR375, FOXM1 protein, Wnt/β-catenin pathway	5-Fu	↑	(10)
	hsa_circ_0000598	miR-340, the BMI1 protein	Irinotecan	↑	(11)
	hsa_circ_0032833	miR-125-5p, MSI1 protein	OXA	↑	(12)
	hsa_circ_0071589	miR-526b-3p, KLF12 protein	DDP	↑	(13)
	hsa_circ_0001806	miR-944, FZD7 protein	DOX	↑	(14)
	hsa_circ_0002211	miR-31-5p, KANK1 protein	5-Fu	↓	(15)
	hsa_circ_0007031	miR-133b, the ABCC5 protein	5-Fu	↑	(16)
	hsa_circ_0000338	miR-217, miR-485-3p	5-Fu	↑	(17)
	hsa_circ_0020095	miR-487a-3p, SOX9 protein	DDP	↑	(18)
multiple myeloma	hsa_circ_0007841	*ABCG2*	DOX	↑	(19)
	hsa_circ_0001141	miR-615-3p, PRKCD protein	BTZ	↓	(20)
	has_circ_0007841	miR-129-5p, JAG1 protein	BTZ	↑	(21)
non-small cell lung cancer	hsa_circ_0007385	miR-519D-3p, *HMGB1*	DDP	↑	(22)
	hsa_circ_103809	miR-377-3p, *GOT1*	DDP	↑	(23)
	hsa_circ_0084003	miR-381-3p, CXCR4 protein	Anti-PD1	↑	(24)
	hsa_circ_0082374	miRNA let-7, *PD-L1*	Anti-PD1	↑	(25)
	hsa_circ_0031250	miR-4458, *REV3L*	DDP	↑	(26)
	hsa_circ_0096157	P21 protein, *CDK4-cyclin D1, Bcl-2*	DDP	↑	(27)
	hsa_circ_0004015	miR-1183, *PDPK1*	TKI	↑	(28)
	hsa_circ_0085131	miR-654-5p, *ATG7*	DDP	↑	(29)
	hsa_circ_0000079	FXR1 protein	DDP	↓	(30)
	hsa_circ_0076305	miR-296-5p, STAT3 protein	DDP	↑	(31)
	hsa_circ_0002483	miR-182-5p, GRB2 protein, FOXO1 protein, FOXO3 protein	PTX	↓	(32)
	hsa_circ_0002130	miR-498, *GLUT1, HK2, LDHA*	Osimertinib	↑	(33)
	hsa_circ_0011292	miR-379-5p, TRIM65 protein	PTX	↑	(34)
	hsa_circ_0014235	miR-520a-5p, *CDK4*	DDP	↑	(35)
	circMTDH.4	miR-630, *AEG-1*	5-Fu	↑	(36)
	hsa_circ_102481	miR-30a-5p, ROR1 protein	TKI	↑	(37)
	hsa_circ_0023303	miR-187-3p, FGF9 protein	DDP	↑	(38)
	hsa_circ_0003998	miR-136-5p, CORO1C protein	DTX	↑	(39)
	hsa_circ_0008928	miR-488, HK2 protein	DDP	↑	(40)
	hsa_circ_0005909	miR-338-3p, SOX4protein	ADM	↑	(41)
	hsa_circ_103615	*ABCB1*	DDP	↑	(42)
	hsa_circ_0072083	miR-195-5p, KPNA4protein	PTX	↑	(43)
	hsa_circ_0014130	miR-493-5p, ROCK1protein	DDP	↑	(44)
	circASXL1	miR-206	DDP	↑	(45)
	hsa_circ_0006404	miR-543, FOXO3 protein	DDP	↓	(46)
	hsa_circ_0003222	miR-527, PHF21B protein, Wnt/β-catenin pathway	Anti-PD1	↑	(47)
	hsa_circ_0002874	miR-1273f, MDM2 protein, p53 protein	PTX	↑	(48)
	hsa_circ_0001658	miR-409-3p, TWIST1 protein	Gefitinib	↑	(49)
	circPRMT5	miR-138-5p, *MYH9*	DDP	↑	(50)
	hsa_circ_0076305	miR-186-5p, *ABCC1*	DDP	↑	(51)
	hsa_circ_0014130	miR-101, *ABCC1*	DDP	↑	(52)
	hsa_circ_0005152	miR-934, SHP2 protein	Anti-PD1	↑	(53)
	hsa_circ_0014130	miR-545-3p, YAP1 protein	DTX	↑	(54)
	hsa_circ_0023404	miR-646, SOX4 protein	DDP	↑	(55)
	hsa_circ_0001821	miR-526-5p, GRK5 protein	PTX	↑	(56)
	hsa_circ_0031608	miR-137, *CXCL12*	DDP	↑	(57)
	hsa_circ_0079587	miR-328-3p, miR-3173-5p, PKP3 protein	Anti-PD1	↑	(58)
	hsa_circ_100565	miR-337-3p, ADAM28 protein	DDP	↑	(59)
lung cancer	hsa_circ_0000199	miR-516b-5p, STAT3 protein	DDP	↑	(60)
	hsa_circ_0030998	miR-558, MMP1 protein, MMP17 protein	PTX	↓	(61)
	hsa_circ_0008717	miR-556-3p, AK4 protein	DDP	↑	(62)
	hsa_circ_0001821	miR-145-5p, ABCC1 protein	DDP	↑	(63)
	hsa_circ_0007798	ASK1 protein	Gefitinib	↓	(64)
hepatocellular carcinoma	hsa_circ_0082002	miR-30-5p, *Snail*, DPP4 protein, *CXCL10*	Anti-PD1	↑	(65)
	hsa_circ_0048677	miR-449c-5p, TIM-3 protein	Anti-PD1	↑	(66)
	hsa_circ_0087293	miR-103a-2-5p, miR-660-3p, Wnt/β-catenin pathway	Sorafenib	↑	(67)
	hsa_circ_0001001	miR-605, FOXO3 protein, ABCB1 protein	OXA	↑	(68)
	hsa_circ_0087293	YBX1 protein	Sorafenib	↑	(69)
	hsa_circ_0104670	miR-155-5p, PDK1 protein	DDP	↑	(70)
	hsa_circ_0031242	miR-924, POU3F2 protein	DDP	↑	(71)
	hsa_circ_G004213	miR-513b-5p, PRPF39 protein	DDP	↓	(72)
	hsa_circ_0025039	miR-1324, MECP2 protein	Sorafenib	↑	(73)
	hsa_circ_0006404	miR-199a-5p, ABCC1 protein	ADM	↑	(74)
	hsa_circ_0003998	miR-218-5p, *EIF5A2*	ADM	↑	(75)
	hsa_circ_0000384	miR-148a, STX3 protein, PTEN protein	DDP	↑	(76)
cervical cancer	hsa_circ_0007874	miR-6893, S100A1 protein	DDP	↑	(77)
	hsa_circ_0023404	miR-5047, VEGFA protein	DDP	↑	(78)
osteosarcoma	hsa_circ_0000073	miR-145-5p, miR-151-3p, NRAS protein	MTX	↑	(79)
	hsa_circ_0001821	ABCB1 protein	ADM	↑	(80)
	hsa_circ_0003496	miR-506-3p, SEMA6D protein, Wnt/β-catenin pathway	DDP	↑	(81)
	hsa_circ_0001141	miR-524, RASSF6 protein	ADM	↓	(82)
	hsa_circ_0081001	miR-494-3p, TGM2 protein	MTX	↑	(83)
	hsa_circ_0005986	miR-760, *EZH2*	DXR	↑	(84)
melanoma	hsa_circ_0020710	miR-370-3p, *CXCL12*	Anti-PD1	↑	(85)
laryngeal cancer	hsa_circ_0004507	miR-873	DDP	↑	(86)
	hsa_circ_0019340	miR-376a, ATG2A protein	DDP	↑	(87)
acute myeloid leukemia	circPAN3	Beclin1, p62, AMPK/mTOR pathway	ADM	↑	(88)
	circPAN3	miR-153-5p, miR-183-5p, XIAP protein	ADM	↑	(89)
thyroid cancer	hsa_circ_0060060	miR-144-3p, *TGF-α*	DDP	↑	(90)
glioma	hsa_circ_0102722	miR-145-5p, ABCG2 protein	TMZ	↑	(91)
	hsa_circ_0005660	miR-132	TMZ	↑	(92)
	hsa_circ_0000284	miR-421, ZIC5 protein	TMZ	↑	(93)
	hsa_circ_0000284	miR-524-5p, KIF2A protein, PI3K/AKT pathway	TMZ	↑	(94)
	hsa_circ_0072083	miR-1252-5p, ALKBH5 protein, NANOG protein	TMZ	↑	(95)
	hsa_circ_0002330	miR-502-5p, NRAS/MEK1/ERK1-2 signal	TMZ	↑	(96)
	hsa_circ_0005198	miR-198, TRIM14 protein	TMZ	↑	(97)
squamous carcinoma of the oral cavity	hsa_circ_0109291	miR-188-3p, ABCB1 protein	DDP	↑	(98)
	hsa_circ_0011946	miR-338-3p, Lin28B protein	DDP	↑	(99)
ovarian cancer	hsa_circ_0001946	miR-1270, the SCAI factor	DDP	↓	(100)
	hsa_circ_0001741	miR-1299, NEK2 protein	PTX	↑	(101)
	hsa_circ_0000714	miR-370-3p, CDK6 protein, RB protein, RAB17 protein	PTX	↑	(102)
	hsa_circ_0063809	miR-1252, FOXR2 protein	PTX	↑	(103)
	hsa_circ_0008234	miR-22, miR-150-3p, *CEBPG, FMNL3*	DDP	↑	(104)
	hsa_circ_0002711	miR-211-5p, HOXC8 protein	PTX	↑	(105)
	hsa_circ_0001756	miR-338-3p, CHTOP protein	DDP	↑	(106)
	hsa_circ_0063809	miR-149-5p, SIK2 protein	PTX	↑	(107)
chronic granulocytic leukemia	hsa_circ_0080145	miR-326, PPFIA1 protein	IM	↑	(108)
	hsa_circ_0009910	miR-34a-5p, ULK1 protein	IM	↑	(109)
bladder cancer	hsa_circ_0000284		GEM	↓	(110)
	hsa_circ_103809	miR-516a-5p, FBXL18 protein	GEM	↑	(111)
	hsa_circ_0001785		DDP	↑	(112)
	hsa_circ_0000285		DDP	↓	(113)
	hsa_circ_0072309	MutSα, ATM, p73 protein	DDP	↓	(114)
	hsa_circ_0008399	WTAP protein	DDP	↓	(115)
prostate cancer	hsa_circ_0006404		DTX	↓	(116)
	hsa_circ_0001427	miR-181c-5p, ARv7 protein	Enz	↓	(117)
	hsa_circ_0000735	miR-7	DTX	↑	(118)
	hsa_circ_0001141	miR-17, Wnt/β-catenin pathway, PI3K/AKT/mTOR pathway	Enz	↓	(119)
	hsa_circ_0005276	miR-1182, TPD52 protein	DTX	↑	(120)
breast cancer	hsa_circ_0025202	miR-197-3p, HIPK3 protein	TAM	↓	(121)
	hsa_circ_0025202	miR-182-5p, FOXO3a protein	TAM	↓	(122)
	hsa_circ_0001839	miR-548p, the PBLD protein	ADM	↓	(123)
	hsa_circ_007874	EG5 protein, TRAF4 protein	Monastrol	↓	(124)
	hsa_circ_0008717	let-7a-5p, the DUSP7 protein	PTX	↑	(125)
	hsa_circ_0001946	miR-7, CCNE1 protein	5-Fu	↑	(126)
	circ-RNF111	miR-140-5p, transcription factor E2F3	PTX	↑	(127)
	hsa_circ_0001667	miR-4458, NCOA3 protein	ADM	↑	(128)
	hsa_circ_0125597	miR-216b, *HMGA2*	5-Fu	↑	(129)
	hsa_circ_0001461	miR-525-5p, SKA1subunit	OXA	↑	(130)
	hsa_circ_0006528	miR-1236-3p, CHD4 protein	ADM	↑	(131)
	hsa_circ_0062558	miR-153-5p, ANLN protein	Lapatinib	↑	(132)
	hsa_circ_0085495	miR-873-5p, integrin β1	ADM	↑	(133)
	hsa_circ_0001598	miR-1184, *PD-L1*	Trastuzumab	↑	(134)
	circ_UBE2D2	miR-200a-3p	TAM	↑	(135)
triple-negative breast cancer	hsa_circ_0005728	miR-512-3p, CDCA3 protein	ADM	↓	(136)
	hsa_circ_0007503	miR-142, WWP1 protein, PI3K/AKT pathway	PTX	↑	(137)
	hsa_circ_005239	miR-361-5p, TLR4 protein	PTX	↑	(138)
renal cell carcinoma	hsa_circ_0035483	hsa-miR-335, *CCNB1*	GEM	↑	(139)
	hsa_circ_0031608	miR-1184, GPCPD1 protein	Sunitinib	↑	(140)
esophageal cancer	hsa_circ_001275	miR-370-3p, WNT7a protein	DDP	↑	(141)
	hsa_circ_0006168	miR-194-5p, JMJD1C protein	PTX	↑	(142)
	circPSMC3	miR-10a-5p, PTEN protein	Gefitinib	↓	(143)
	hsa_circ_0000337	miR-377-3p, JAK2 protein	DDP	↑	(144)
gastric cancer	hsa_circ_0026359	miR-1200, the POLD4 protein	DDP	↑	(145)
	hsa_circ_0006990	miR-125b-5p, STAT3 protein	DDP	↑	(146)
	hsa_circ_0032821	miR-515-5p, SOX9 protein	OXA	↑	(147)
	hsa_circ_0000234	miR-142-3p, ROCK2 protein	DDP	↑	(148)
	hsa_circ_0058147	miR-182-5p	DDP	↑	(149)
	hsa_circ_0001313	miR-618, BCL2protein	DDP	↑	(150)
	hsa_circ_0000199	miR-198, PIK3R1 protein, p85α protein, PI3K/AKT pathway	DDP	↑	(151)
	hsa_circ_0000657	miR-99a-5p, the MTMR3 protein	DDP	↓	(152)
	hsa_circ_0001821	miR-124-3p, ZEB1 protein	PTX	↑	(153)
	hsa_circ_0004771	miRNA-138-5p, HIF-1α factor	5-Fu	↑	(154)
	hsa_circ_0006089	miR-330-3p, NT5E protein	DDP	↑	(155)
	hsa_circ_0004339	miR-802, *BMI1*	DDP	↑	(156)
	circ MTHFD2	miR-124, *MDR-1*	MTA	↑	(157)
	hsa_circ_0001546	miRNA-421, ATM/CHK2/P53 pathway	OXA	↓	(158)
	hsa_circ_0031452	miR-137, PBX3 protein	DB	↑	(159)
	hsa_circ_0000144	miR-502-5p, ADAM9 protein	OXA	↑	(160)
	hsa_circ_0000260	miR-129-5p, MMP11 protein	DDP	↑	(161)
	hsa_circ_0001821	miR-152-3p, HDGF/PI3K/AKT pathway	DDP	↑	(162)
	hsa_circ_0001821	miR-30a-5p, YAP1protein	DDP	↑	(163)
	hsa_circ_0002570	miR-490-3p, HMGA2protein, HNRNPK protein, β-catenin	DDP	↑	(164)
pancreatic	hsa_circ_0000284	miR-330-5p, RASSF1 protein	GEM	↑	(165)
	hsa_circ_0013587	miR-1227, E-Cadherin	Erlotinib	↓	(166)
	hsa_circ_0030167	miR-338-5p, Wif1, Wnt8, β-catenin	GEM	↑	(167)

↑ means increased; ↓ means decreased.

## CircRNAs Regulate Tumor Drug Resistance by Affecting Intratumor Drug Concentrations

It has been shown that reduced drug concentration is the main cause of drug resistance. This may be due to drug segregation in intracellular vesicles and compartments, increased drug efflux or decreased drug influx. These changes may be due to the remodeling of drug channels and transporters, which broadly include ATP-binding cassette (ABC) family proteins, solute carriers, and volume-regulated anion channels (VRACs). M Saxena et al. mechanistically demonstrated that the promoters of ABC transporter proteins carry binding sites for several EMT-inducible transcription factors and that EMT-related transcription factors, such as Twist, Snail, and FOXC2, and overexpression of these transcription factors increased the promoter activity of ABC transporter proteins. Furthermore, chromatin immunoprecipitation studies showed that Twist binds directly to the E-box element of the ABC transporter protein. EMT inducers were therefore identified as novel regulators of ABC transporter proteins, thus providing a long-term link between aggressiveness and multidrug resistance. Thus, targeting EMT transcription factors could serve as a novel strategy to inhibit tumor metastasis and associated drug resistance (168–171).

According to current reports, circRNAs can increase drug resistance in tumor cells by targeting miRNAs and down-regulating the expression of genes related to drug concentrations in tumors accordingly. For example, in glioma tissues and cell lines, circRNA CEP128 expression was upregulated, and circRNA CEP128 expression was higher in temozolomide-resistant glioma cells than in their parental cells. In contrast, downregulation of circRNA CEP128 inhibited glioma cell proliferation and reduced temozolomide resistance by decreasing the expression of ATP-binding cassette G superfamily member 2 (ABCG2). In addition, miR-145-5p was expressed at low levels in glioma cells and temozolomide-resistant glioma cells, and miR-145-5p was also identified as a target of circRNA CEP128. miR-145-5p overexpression inhibited the proliferation of U251/temozolomide cells and decreased the expression of ATP-binding cassette superfamily member 2, and circRNA CEP128 overexpression blocked these changes (91).

The mRNA and protein levels of ATP-binding cassette transporter protein G2 (ABCG2) were elevated in myeloma adriamycin chemotherapy-resistant cells. Silencing hsa_circ_0007841 in resistant cells reduces the mRNA and protein levels of ABCG2, and re-expression of hsa_circ_0007841 blocks this reduction. Similarly, overexpression of hsa_circ_0007841 effectively upregulated mRNA and protein levels of ABCG2. Inhibition of ABCG2 blocked hsa_circ_0007841 overexpression-induced cellular chemoresistance. In addition, ABCG2 reduced the difference in the half-maximal inhibitory concentration between parental and resistant cells. These findings suggest that hsa_circ_0007841 can promote a pattern of acquired chemoresistance in myeloma cells through the upregulation of ABCG2, providing a new molecular basis for chemotherapy in multiple myeloma (19).

In laryngeal cancer, knockdown of circ_0004507 reduces the protein levels of multidrug resistance-associated protein-1 (MRP1) and multidrug resistance gene 1 (*MDR1*), which are associated with cellular cassette transporters and confer multidrug resistance to tumor cells by reducing the uptake of anticancer drugs (86).

**Figure 2:**
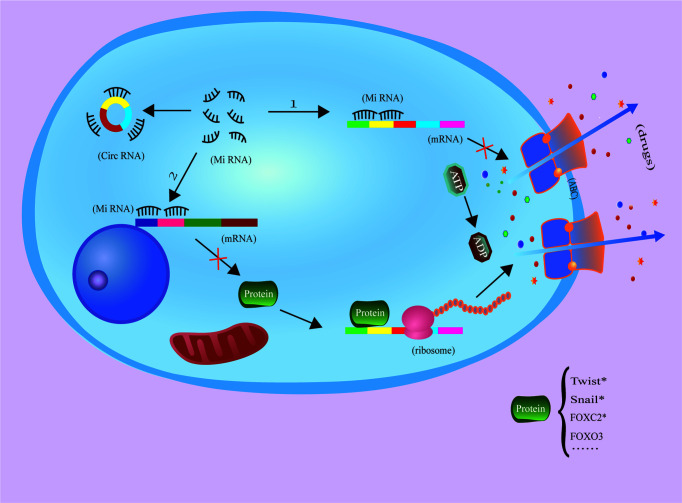
CircRNAs inhibit miRNA function throug1h the molecular sponging mechanism, increasing ABC expression and drug efflux to increase tumor cell drug resistance. The protein with * has been proven to be an ABC-related transcription factor, but there is no literature to prove that it is regulated by circRNA.

In hepatocellular carcinoma, circRNA PTGR1 regulates drug resistance to 5-fluorouracil (5-FU) and the growth tumor cells by regulating the miR-129-5p/ABCC1 axis (172). Additionally, studies of adriamycin resistance in hepatocellular carcinoma have shown that circFOXO3 promotes resistance to adriamycin in by regulating the miR-199a-5p/ATP binding cassette C family member 1 axis (74). However, circRNA circFBXO11 regulates hepatocellular carcinoma progression and oxaliplatin resistance through the miR-605/FOXO3/ABCB1 axis (68), so further study is needed determine whether circFBXO11 regulates circFOXO3 and its downstream signaling through this axis (**Figure 2**).

It can be seen that the effect of circRNA on ABC transporter protein is important and diverse in each type of tumor, but there are various factors that affect the concentration of drugs in tumor cells, and ABC transporter protein is only one of the drug channels. However, it is worth thinking why circRNA are mostly reported to regulate ABC transporter protein.

## CircRNAs Enhance EMT and Stemness

Briefly, EMT is an epigenetic program by which cells lose their epithelial phenotype and acquire mesenchymal features. Common EMT-inducible transcription factors, such as twist and snail, which contain complementary binding sites, act as promoters of ABC transporters, and they enhance not only EMT but also ABC transporter expression (4, 171). In recent years, it has been shown that circRNAs can influence tumor cell resistance by enhancing EMT; for example, circ_001680 and circ-NOTCH1 are elevated in colorectal cancer (CRC) and gastric cancer (GC), respectively, and both these circRNAs can promote cancer stem cell (CSC) expansion *via* EMT (11, 173). Chen et al. showed that failure to eradicate cancer cells that acquire stem cell features through EMT activation or senescence is another reason for impaired cancer treatment outcomes (174–177). Second, CSCs are able to remain dormant for a long period of time to escape harmful stresses such as radiotherapy and chemotherapy. Once harmful stress is lifted, CSCs can differentiate, proliferate or metastasize at the primary lesion and subsequently invade other organs, leading to tumor recurrence or metastasis (178, 179). For example, circFOXO3 reduction promotes prostate cancer progression and doxorubicin resistance. circFOXO3 inhibits prostate cancer cell survival, migration, invasion, and doxorubicin resistance, which is associated with circFOXO3-mediated inhibition of Foxo3 and EMT (116).

**Figure 3:**
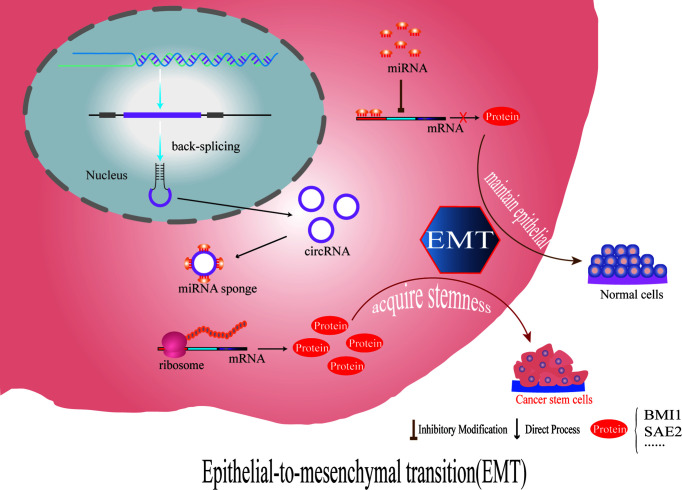
CircRNAs regulate the stem cells of tumor cells by affecting the expression of EMT- and stemness-related proteins, which leads to chemotherapy resistance.

Nithila A. Joseph et al. showed that in lung adenocarcinoma cells, circRNA CCDC66 increases SUMOlyation modifications related to the HGF-MET signaling pathway and epidermal growth factor receptor (EGFR)-controlled SAE2 through association with EMT and thus affects the resistance of tumor cells to tyrosine kinase inhibitors (TKIs) targeting EGFR (180). Among them, SAE2 is one of the subunits of SUMO-activating enzyme E1, and SUMO-mediated modifications have been shown to be importantly associated with the proliferation, migration and invasion of a variety of tumors (181, 182). We found that circRNA CCDC66 has been reported to be associated with mechanisms of chemoresistance generation not only in lung adenocarcinoma cells but also in gastrointestinal tumors; for example, DHX9 phosphorylation induced by oxaliplatin chemotherapy in colorectal cancer promotes the expression of oncogenic circRNA CCDC66 to affect chemoresistance (183), and circRNA CCDC66 in gastric cancer through the miR-618/BCL2 axis regulates cisplatin resistance in gastric cancer (150). More data is needed to assess the resistance induced by different mechanisms of different drugs yet related to the same circRNA (**Figure 3**).

It is thus clear that circRNA also has an important role in the regulation of EMT and stemness. However, EMT and stemness is different from the regulation of drug channel proteins, and changes often imply an all-round overall effcet in EMT and stemness. Therefore, it is clear that since circRNA can enhance drug resistance by regulating EMT and stemness, circRNA must have other regulatory functions while enhancing EMT and stemness. In short, the effect of circRNA on tumors, too, is holistic. For example, the circ-CPA4 regulated cell growth, mobility, stemness and drug resistance in NSCLC cells and inactivated CD8 T cells in the tumor immune microenvironment (25).

## CircRNAs Alter the Copy Number of Target Genes and Activate Bypass Pathways or Downstream Signaling

### CircRNAs Compete for Endogenous MiRNAs

CircRNAs competitively bind miRNAs (184, 185), so as to serve as intracellular competitive endogenous RNA, suppressing the effects of miRNAs on target genes. For example, the circRNA AKT3 enhances cisplatin resistance in gastric cancer by inhibiting miR-198 and upregulating PIK3R1, which was experimentally shown to be a downstream molecule of miR-198 (151). PIK3R1 encodes a regulatory subunit of PI3K and has a role in positively regulating the PI3K signaling pathway. The autophagic process also requires phosphatidylinositol 3-phosphate (PI3P), and the inhibition of PI3P can inhibit the formation of autophagic vesicles. Whether PI3K can affect tumor cell drug resistance through the regulation of autophagy deserves further study.

In addition, some studies have shown that circRNA can promote its own loop and form positive feedback by up-regulating the expression of downstream transcription factors. For example, in gastric cancer, circFAM73A increases the expression of HMG (high mobility group) A2 through miR-490-3p, HMGA2 plays a dichotomous role in regulating circFAM73A ex-pression, i.e., HMGA2 facilitates the transcriptional acti-vation of FAM73A by E2F1 and elevates the efficiency of circFAM73A circularization by HNRNPL, HMGA2 promotes stem cell-like properties and cell malignancy in GC cells (164). It is worth considering whether this process of positive feedback loops to improve tumor drug resistance is common. With regard to circRNA and its sponge miRNA, through a large number of literature review and summary, we find that there is often more than one miRNA combined with each other under the same circRNA. For example, also in non-small cell lung cancer, circRNAPRMT5 can target miR-381-3p (24) and miR-4458 (26) to regulate the expression of different downstream proteins to promote tumor, and the same circ_007630 can also target miR-296-5p (31) and miR-186-5p (51) respectively. Interestingly, the same miRNA targets different genes in different tumors and regulates downstream pathways, such as miR-338-3p, which promotes the progressive drug resistance of oral squamous cell carcinoma (99) and ovarian cancer (106) at the same time. As we all know, the effect of circRNA on tumor drug resistance is often the result of a variety of mechanisms, so we need to pay attention to the interaction between various mechanisms and pathways, and pay attention to the integration and analysis of various mechanism information. The coincidence targets in these different pathways provide some support for us to analyze the generation of drug resistance of tumor cells as a whole. For example, miR-338-3p can play a role in promoting drug resistance in a variety of tumors according to current reports (99, 106), but the mechanisms of action are different. It is worth thinking whether the regulatory mechanisms of the same miRNAs in different tumor lines overlap, and if not, what determines the target of miRNAs to exert regulatory effects in tumor cells, and if so, whether it is of limited significance to focus on individual pathways according to most of the current literature. Moreover, we found that there are literatures about target overlap at all three levels of circRNA, miRNA and protein (32, 51, 63, 88, 89, 149).

### CircRNAs Function as a Reservoir or a Stabilizing Factor for the MiRNA

Studies have shown that in the progress of chemoresistance of prostate cancer, as circRNA17 is positively correlated with the expression level of miR-181c-5p, this circRNA may not function as a sponge to sequester the miRNAs, but likely function as a reservoir or a stabilizing factor for the miR-181c-5p (117). Mechanistically, speculated the binding of miR-181c-5p with circRNA17 might enhance the miR-181c-5p stability by repressing its degradation through nucleases such as Tudor-SN endonuclease (186). In addition, this stabilizing effect appears particularly significant *in vivo* during tumor growth and less so in cells grown in tissue culture. The exact details of these phenomena remain to be further determined. CircRNA adsorbs miRNA can prevent the degradation of the latter, which reminds us of the need to add deeper thinking to the traditional study of ceRNA(competing endogenous RNA) mechanism.

### CircRNAs Bind RNA-Binding Proteins

CircRNAs serve as protein baits or antagonists, thus arrestting the function of proteins, thereby affecting the related progresses (184). For example: circLIFR synergizes with MSH2 to attenuate chemoresistance *via* MutSα/ATM-p73 axis in bladder cancer (114). In addition, the same circRNA can perform different functions in different cells or in the same cell through different pathways. For example, in hepatocellular carcinoma cells, circRNA-SORE can mediate hepatocellular carcinoma resistance to sorafenib by stabilizing YBX1 (67), while N-6-methyladenosine-modified circRNA-SORE can also maintain hepatocellular carcinoma resistance to sorafenib by regulating β-catenin signalin (69). It should be pointed out that the previously mentioned circRNA-SORE mediates the drug resistance of hepatocellular carcinoma to sorafenib by stabilizing the YBX1 protein; in fact, it does not involve gene copy number and activation, but rather, circRNA directly plays a role in the process of protein stability and degradation.YBX1 is a kind of RNA binding protein (RBP). At present, an increasing number of evidences suggest the direct binding of circRNAs to RBPs. Most of them continue to regulate the expression of downstream signaling pathways by affecting the stability or degradation of target proteins, and there is often more than one downstream pathway involved (30, 124). Although the RBP pathway is different from pathways influenced by circRNA regulation of the target gene, in essence, it continues to play its biological function by regulating the expression (by targeting genes) or degradation (by targeting proteins) of related proteins.It is worth mentioning that there are also reports that circRNA directly binds to the DNA promoter to regulate transcription and thus affect the progression of liver cancer (84, 187).

### CircRNAs Encode Proteins

**Figure 4:**
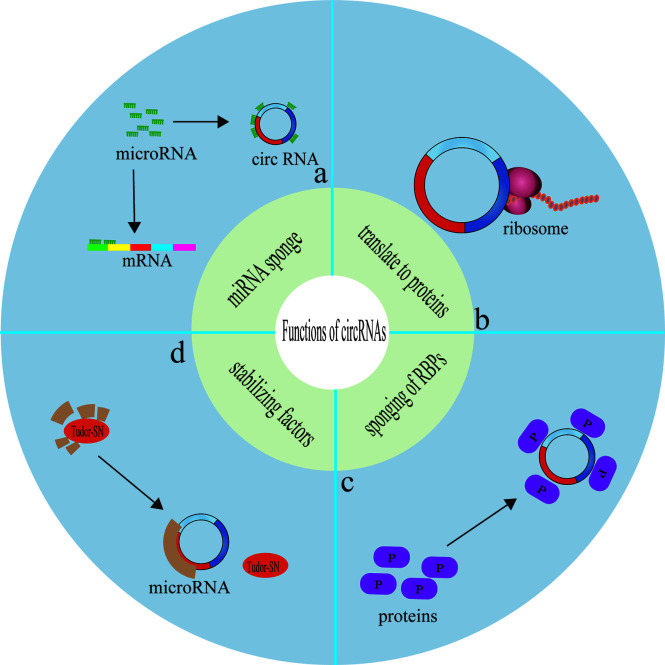
Functions of circRNAs. **(A)** circRNAs competitively bind miRNAs, so as to serve as intracellular competitive endogenous RNA, suppressing the effects of miRNAs on target genes. **(B)** circRNAs can translate to proteins through a cap-independent manner. **(C)** circRNAs serve as protein baits or antagonists, thus arrestting the function of proteins, thereby affecting the related progresses. **(D)** circRNA function as a reservoir or a stabilizing factor for the miRNA.

CircRNAs can translate to proteins through a cap-independent manner (184, 188). Yang et al. demonstrated that N-methyladensine(m6A) could promote the initiation of protein translation from circRNAs in human cells (189). For example, PengLi et al. found that circMRPS35 not only regulates the STX3-PTEN axis through sponge miR-148A, but also encodes circMRPS35-168aa, resulting in cisplatin resistance, thus playing a carcinogenic role in hepatocellular carcinoma (76). In addition, in gastric cancer, YuZhang et al. found that circDIDO1 inhibits the progression of gastric cancer by encoding a new DIDO1-529aa protein and regulating the stability of PRDX2 protein (190). At present, there are relatively few articles about circRNA affecting tumor chemotherapy resistance by encoding proteins, and in most of these articles, circRNA plays a role in multiple pathways, For example, even playing the role of molecular sponge also encodes protein, the emergence of this experimental result may be preconceived thinking about the mechanism of circRNA molecular sponge. Whether this preconceived way of thinking limits the study of circRNA multi-mechanism is worthy of further study (**Figure 4**).

Although this section outlines that circRNAs affect cancer drug resistance through the copy number alteration and activation of downstream genes, circRNA downstream genes are often proto-oncogenes or oncogenes, and the outcomes are mostly achieved by affecting the immune escape of cancer cells and the ability of cancer cells to repair genes or altering autophagy or EMT. For example, in studies of drug resistance in non-small-cell lung cancer (NSCLC), circFGFR1 also promotes NSCLC cell development and immune system escape by sponging miR-381-3p and targeting CXCR4, a gene that has been identified as a key oncogene in several cancers, and knockdown of CXCR4 reverses circFGFR1-induced NSCLC cell proliferation, migration and invasion (24). In addition, circFGFR1 is a sponge for several miRNAs. Additionally, in hepatocellular carcinoma cell resistance-related studies, circMET,can drive hepatocellular carcinoma immunosuppression and anti-PD-1 therapy resistance *via* the miR-30-5p/Snail/DPP4 axis. circMET also induces hepatocarcinogenesis and immune tolerance *via* the Snail/DPP4/CXCL10 axis (65).

The role of circRNAs in tumor drug resistance can be broadly classified into two categories according to their role, and in addition to the drug resistance enhancing effect mentioned above, there are also reports of drug resistance inhibiting effects. CircRNA can be negatively correlated with the generation of tumor drug resistance,For example, circ_0000079 induces the ribonucleic acid binding protein FXR1 to block the formation of the FXR1/PrCKI complex and reduces its promoting effects on NSCLC cell invasion and cisplatin resistance; it was shown that circ_0000079 (CiR79) levels were significantly downregulated in NSCLC patients, especially in cisplatin-resistant NSCLC patients, and that reduced circ_0000079 levels were significantly associated with overall survival in NSCLC patients. The results of Cell Counting Kit-8 and Transwell cell invasion assays showed that circ_0000079 overexpression significantly inhibited the proliferation and invasion of cisplatin-resistant NSCLC cells. Functionally and mechanistically, circ_0000079 negatively regulates the FXR1/PRKCI-mediated phosphorylation of glycogen synthase kinase 3β and activator protein 1, thereby decreasing the protein levels of the *Snail* gene, an important promoter gene that regulates cancer cell growth and EMT (30). This shows that circRNAs may be able to exert opposite effects on the biological behavior of tumor cells through different signaling pathways in different tumor cells.

## CircRNAs Regulate Endoplasmic Reticulum Stress, Autophagy and Phagocytosis

Both the activation and inhibition of endoplasmic reticulum stress have been shown to attenuate chemo- and radioresistance in tumors (191–193), and endoplasmic reticulum stress is also a potent trigger of autophagy for controlling cancer sensitivity (194). Autophagy is a process by which cells engulf their own cytoplasmic proteins or organelles and encapsulate them into vesicles that fuses with lysosomes to form autophagic lysosomes, which degrade the contents they encapsulate. However, autophagy is also a double-edged sword. Mild autophagy facilitates cell survival under normal or mild stress conditions, while excessive autophagy can lead to cell death. Smith et al. showed that the development of drug resistance may be attributed to autophagy-mediated oxidative stress and the role of key metabolites provided by autophagy in maintaining cell stemness during dormancy (195–198).

However, in contrast to other mechanisms of drug resistance generation, autophagy and phagocytosis tend to be positively correlated with the generation of drug resistance, i.e., enhanced autophagy generates drug resistance and inhibition of autophagy inhibits drug resistance, presumably related to the specificity of the function of autophagy itself. In cervical cancer, for example, autophagy-related genes are aberrantly regulated in cells overexpressing hsa_circ_0023404 (78). Nevertheless, in studies of drug resistance in cervical cancer, circMTO1 can regulate the expression of the S100A1 protein *via* miR-6893 and can likewise affect the expression of autophagy-related genes (77).

In laryngeal cancer, circPGAM1 can enhance autophagic signaling during drug resistance by regulating miR-376a, and autophagy can be induced and activated by autophagy-related genes in light chain 3 (LC3). Functionally, autophagy plays a dual role by promoting cell death and cancer cell survival (87).

In acute myeloid leukemia (AML), circPAN3 is involved in AML drug resistance by regulating autophagy. The study suggests that circPAN3 may promote AML drug resistance by regulating autophagy and affecting the expression of apoptosis-related proteins, while AMPK/mTOR signaling plays a key role in circPAN3 regulation of autophagy. Moreover, there is more than one miRNA strongly predicted to be associated downstream of circPAN3 in this example, and regulating autophagy *via* the AMPK/mTOR pathway may also be only one of these pathways; whether other more precise downstream pathways of circPAN3 exist to influence tumor drug resistance remains to be investigated (88). Similarly, in studies of AML drug resistance, circPAN3 was also found to mediate AML drug resistance *via* the miR-153-5p/miR-183-5p-XIAP axis (89). Therefore, by summarizing the studies of autophagy-related circRNAs inducing drug resistance, we found that drug resistance induced by the regulation of autophagy and phagocytosis is often not mediated by a single mechanism of action.

**Figure 5:**
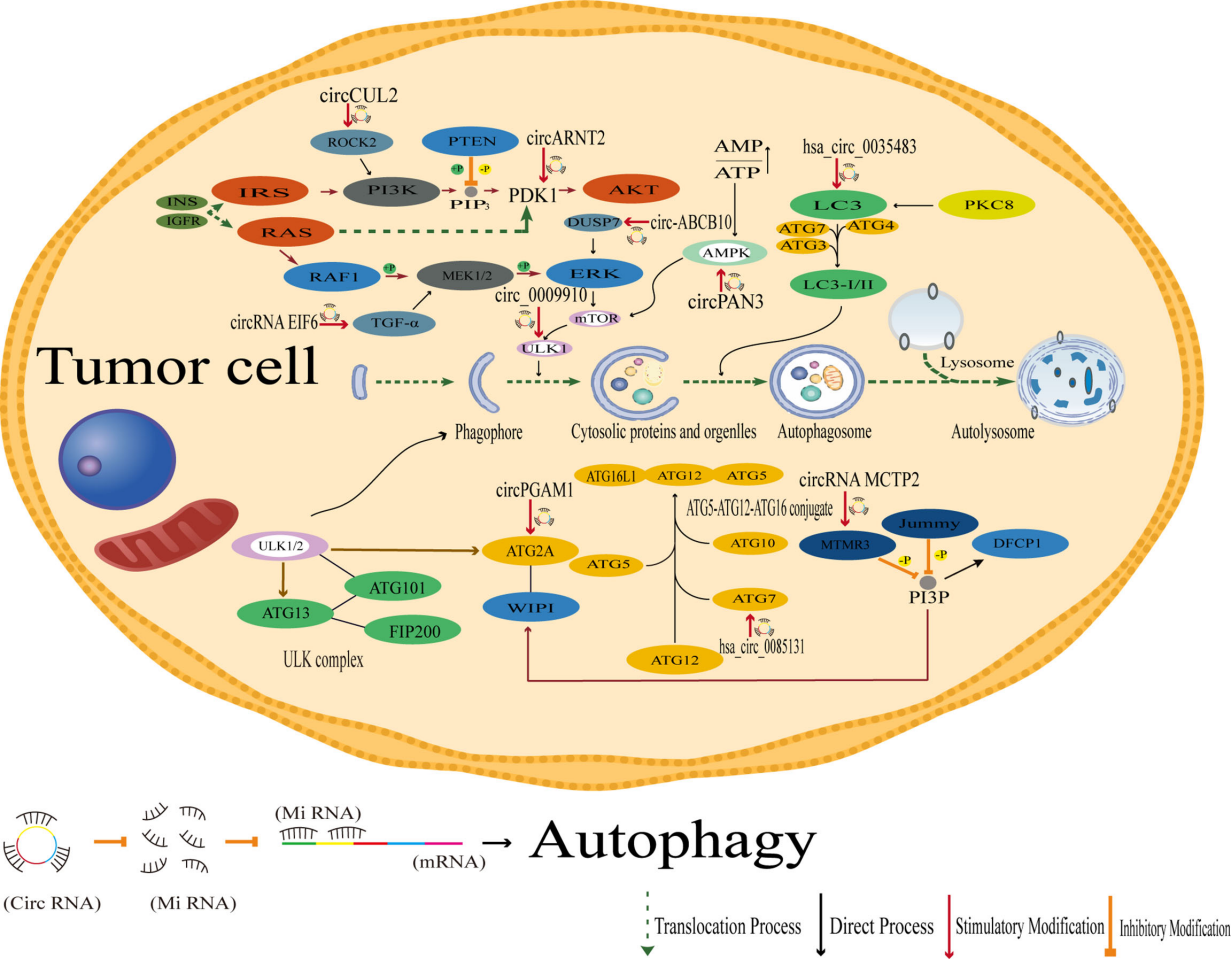
CircRNA regulate autophagy-mediated chemoresistance.

Some examples of inhibiting drug resistance by suppressing autophagy are as follows: in a study of drug resistance in gastric cancer, Sun et al. found that circRNA MCTP2 inhibited cisplatin resistance in gastric cancer through miR-99a-5p-mediated MTMR3 expression, and mechanistically, miR-99a-5p directly targeted MTMR3, a myotubulin-associated protein, to inhibit autophagy (152). mTMR3 is a member of the myotubulin family. It is an inositol-like 3-phosphatase that hydrolyzes PtdIns3P (PI3P) (199). PI3P (inositol-like 3-phosphate) is required for the autophagic process, and the inhibitory effect of MTMR3 on PI3P inhibits the formation of autophagic vesicles. In this study, the action of the circRNA MCTP2 molecular sponge led to the inhibition of autophagy through the MTMR3 protein, which then caused changes in related proteins that regulate autophagy, such as p62, related genes, such as BCL-2 and Beclin1. The process of Sun et al. provides ideas to study the regulation of autophagy, but it is unknown whether there is an inverse regulatory relationship between the regulatory proteins or genes and the functional units of autophagy (**Figure 5**).

## CircRNAs Promotion or Inhibition of DNA Repair

The induction of DNA damage is another major effect of radiotherapy and chemotherapy, usually leading to cell death. However, DNA repair can reduce the sensitivity of cancer cells to treatment (4), so enhancing or suppressing DNA repair can affect the proportion of tumor cells killed by drugs and, in turn, affects drug resistance. For example, in gastric cancer, it has been shown that circ_0026359 is overexpressed and correlated with cisplatin resistance and poor survival in gastric cancer patients, and conversely, deletion of circ_0026359 enhances miR-1200 activity, which reduces the expression level of POLD4 and thus reduces the resistance of gastric cancer cells to cisplatin. In contrast, POLD4 plays a role in cell proliferation and the maintenance of human cell genome stability. Low expression of POLD4 has been reported to impair DNA repair systems, including nucleotide excision repair, and increase the risk of lung cancer formation (145). As an additional example, in triple-negative breast cancer, hsa_circ_0000199 may activate the DNA repair molecule histone family member X (H2AX), leading to cisplatin resistance. This also involves the classical PI3K/AKT pathway (151, 200).

CircRNA AKT3 upregulates PIK3R1 by inhibiting miR-198 to enhance cisplatin resistance (by inhibiting apoptosis and facilitating DNA damage repair) in gastric cancer. Mechanistically, circAKT3 activates the PI3K/AKT signaling pathway in GC cells by sponging miR-198 to eliminate the inhibitory effect of this miRNA on its target gene *PIK3R1*. Activation of the PI3K/AKT pathway contributes to the upregulation of the DNA repair molecule BRCA1, leading to resistance to cisplatin-based chemotherapy.CircAKT3 affected the DNA damage response (DDR) in GC cells, suggested that circAKT3 may enhance cisplatin resistance through the PI3K/AKT pathway and DDdR mechanism in GC cells (151). In summary, to study the effect of DNA repair ability on tumor drug resistance, it is crucial to determine the connection between circRNA and DNA damage repair genes or proteins, which are commonly associated with DNA damage repair, such as POLD4, H2AX, BRCA1, etc. In addition, these genes or proteins are often linked to some classical signaling pathways, probably because repair involving DNA requires some well-established and stable genes and pathways.

## CircRNAs Regulate Tumor Microenvironment (TME)

The TME is a complex ecosystem with microbiota, an acidic pH, inflammatory factors, matrix metalloproteinases, extracellular matrix (ECM), tumor-associated fibroblasts (CAFs), and tumor-associated macrophages (TAMs). The impact of the tumor microenvironment on the tumor is frequent and must be considered, for example, because the TME of the primary tumor and the corresponding metastases differ, which affects the sensitivity of cancer cells (201, 202). It has been demonstrated that external factors such as inflammatory factors, acidity and alkalinity of the microenvironment in which the tumor is located regulate tumor drug resistance by influencing protein synthesis, organelle division, and enzyme function in tumor cells. The TME can both directly and indirectly affect the ability of circRNAs to regulate tumor drug resistance; for example, the inflammatory factor IL-7 can reduce the expression of ABCG2 (ATP-binding cassette family protein) and regulate cisplatin resistance in NSCLC (203). A hypoxic tumor microenvironment can cause excessive mitochondrial division of NK cells, which reduces the immune surveillance function of the organism (204). Direct regulation of circRNAs is also possible; for example, in a study on the previously mentioned circRNA CCDC66 in lung adenocarcinoma resistance, the expression of SAE2 was mainly regulated by EGFR, whereas the expression of circRNA CCDC66 was positively regulated by FAK and c-Met and negatively regulated by nAchR7α. The immediate response to hypoxia can increase phosphorylated c-Met and SAE2 expression and EMT (180). This regulates tumor drug resistance upstream from circRNA. It is thus clear that the TME and the role of circRNAs are closely related, and the effect of the TME on tumor drug resistance may bring new ideas and possibilities for the exploration of mechanisms for drug-resistant tumors in circRNA research.

## CircRNAs in Immune Checkpoint Inhibitors Resistance

Chemotherapeutic drugs have no specific target, and their effects are all over the body, and they are extremely sensitive to cells that proliferate rapidly. It can inhibit and kill the rapidly proliferating tumor cells, but also inevitably “accidentally” injure the normal cells of human body, which is a simple and brutal anti-cancer method of “Injure the enemy by 1000 and lose 800 by yourself”. In contrast, there are sites on the surface of targeted drugs that can specifically bind to tumor cells, which can precisely identify the lesion site, and when cancer cells are identified by immune checkpoint inhibitors, they are destroyed. The effect on normal cells is minimal. Immune checkpoint inhibitors (ICIs) have been shown to be highly efficient in the treatment of solid tumors, Therefore, ICIs treatment may be an important tool for tumor treatment in the future. However, limited benefit in terms of response and survival has been reported in many patients because of drug resistance. PD-L1 expression remains the only validated marker in clinics, molecular profiling has brought valuable information, showing that the tumor mutation load and microsatellite instability (MSI) status were associated to higher response rates in nearly all cancer types (205). The main immune checkpoint inhibitors currently in clinical use are anti-CTLA-4 and antibodies against PD-1 and its ligand PD-L1, which can be applied to many types of cancer and have shown significant improvements in patient survival time. However, despite the success of ICIs, resistance to these agents restricts the number of patients able to achieve durable responses, and immune-related adverse events complicate treatment. Due to tumor heterogeneity, current limited research shows that PD-1 or PD-L1 monoclonal antibody drug resistance may be related to the following factors: mutation of tumor antigen and antigen presentation process, multiple immune checkpoint interactions, immune microenvironment changes dynamically, activation of oncogenic pathways, gene mutation and epigenetic changes of key proteins in tumors, tumor competitive metabolism, and accumulation of metabolites, etc, mechanisms of resistance are complex. Therefore, it is the most urgent task to further elucidate the mechanism of immune checkpoint inhibitor resistance, discover multitumor universal biomarkers, and develop new target agents to improve the response rate of immunotherapy in patients (206). It has been shown that there is also a strong link between circRNAs and ICIs treatment resistance. Zhang et al. found circFGFR1 could directly interact with miR-381-3p and subsequently act as a miRNA sponge to upregulate the expression of the miR-381-3p target gene C-X-C motif chemokine receptor 4 (CXCR4), which promoted NSCLC progression and resistance to anti-programmed cell death 1 (PD-1)- based therapy in non-small cell lung cancer cells (24). Interestingly, Hong et al. found that knock-down of circ-CPA4 inhibited intracellular and extracellular PD-L1 by targeting let-7 miRNA. On the one hand, PD-L1 self-regulated NSCLC cell growth, mobility, stemness and chemoresistance to cisplatin treatment. On the other, secreted PD-L1 inactivated CD8 T cells by activating extracellular and intracellular pathways mediated cell death to facilitate immune evasion (25). There are many more reports on circRNAs regulating resistance to ICIs, mainly against PD-1 and PD-L1 in breast cancer and hepatocellular carcinoma (65, 134). CircRNAs regarding anti-CTLA-4 resistance are rare by comparison, but there are reports of circRNAs targeting CTLA4 *via* ceRNA(competing endogenous RNAs) mechanisms to regulate tumor tumorigenesis (207, 208). Since ICIs treatment is closely related to T cell immunity and tumor microenvironment, and circRNA also plays an important role in this regard, I think the possible relationship of circRNA in future ICIs treatment cannot be ignored.

## Conclusion and Perspective

**Figure 6:**
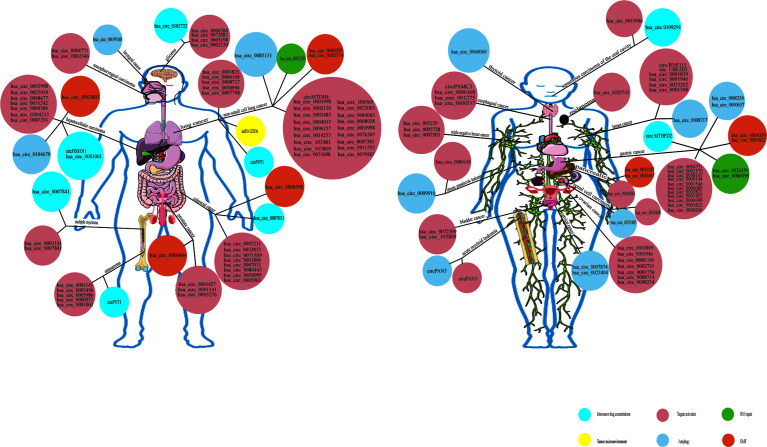
Overview of functional circRNAs in various types of cancer. The map shows the circRNAs that have been confirmed to function in various types of cancer. As the mechanism of drug resistance is complex, only the main mechanism is listed in this map. Among the number other mechanisms, specific mechanisms such as the regulation of metabolism, cell cycle, apoptosis, RNA binding proteins (RBPs), immune escape,etc, summed up as alterations in target gene copy number and the activation of bypass pathways or downstream signaling.

Tumor resistance is now known to be facilitated by factors affecting increased drug efflux transporters or reduced influx channels, enhanced EMT and stem cellularity, alterations in target gene copy number and the activation of bypass pathways or downstream signaling, endoplasmic reticulum stress, the remodeling of autophagy and phagocytosis, enhanced or diminished DNA repair capacity, and alterations in the TME (**Figure 6**). This paper summarizes the links between the above mechanisms and circRNAs. From the beginning, circRNA was considered to be the “junk” in the cell, Research in recent years has discovered functions of circRNAs ranging from being microRNA (miRNA) sponges and transcription regulators to having protein in teractions and allowing for translation. The function and mechanism of action of circRNAs in tumors is becoming increasingly complex, especially after considering again the function of circ’s encoded proteins, the binding of a previously large number of circRNAs to downstream targets does not seem to be so pure. This also predetermines the diversity of circRNAs functions, in this case, the integration of the same circRNAs function is particularly important. In addition to the functional complexity, the function of the same circRNAs in different tumors or different environments is still worth thinking about whether the function of circRNAs will change with the cellular environment. Secondly, regarding the characterization of circRNAs distribution in cells, researchers habitually take the site where circRNAs saccumulates more (nucleus or cytoplasm) as the site where circRNAs exerts its function, and whether this is reasonable deserves further consideration. Because, we know that the content of circRNAs varies greatly in different tumors, the circRNAs enriched in a certain type of tumor, even if it is relatively less distributed, may still be quite higher after quantification than in other cells where it is more distributed. The role of circRNAs in tumor cells is also becoming increasingly important, and there exists a great potential to become a molecular marker and therapeutic target for tumors in the future. It is believed that, based on the complex mechanism of drug resistance, it is of no clinical significance to simply search for and regulate drug resistance targets, and it may even produce drug resistance again soon. It is speculated that according to the possible tumor characteristics, three types of treatment methods should be combined to change the tumor microenvironment ecology and eliminate various heterogeneous tumor subsets, so as to reduce tumor drug resistance and improve long-term clinical efficacy. Finally, standardizing the nomenclature of circRNAs in publications is imminent. The traditional way of naming circRNAs based on the parent gene has many drawbacks, because it is impossible to distinguish different circRNAs composed of different exons under the same parent gene, and even in many circRNAs related to tumor drug resistance articles do not provide the sequence or location of circRNAs, and the simple parent gene naming makes it impossible for me to target the exact circRNA. Of course, this problem is inevitable, because database inclusion and naming always follows the publication of the researcher’s results. I suggest that the first discovered circRNA can follow the nomenclature of the publisher, but subsequent articles need to specify the sequence and composition of the circRNA to distinguish if it is the same circRNA.

## Author Contributions

SW, LQ, and TC collected the related reports and drafted the manuscript. XH, YX, and LX revised the manuscript. YJ, HH, QF, QL, YW, and JW participated in designing the review. All authors contributed to the article and approved the submitted version.

## Funding

This work was supported by the National Natural Science Foundation of China (81902515), Excellent Youth Talent Support Program (Key) of Anhui Province (gxyqzd2020029), and Introducing Talents Natural Science Foundation of The First Affiliated Yijishan Hospital of Wannan Medical College (YR202006).

## Conflict of Interest

The authors declare that the research was conducted in the absence of any commercial or financial relationships that could be construed as a potential conflict of interest.

## Publisher’s Note

All claims expressed in this article are solely those of the authors and do not necessarily represent those of their affiliated organizations, or those of the publisher, the editors and the reviewers. Any product that may be evaluated in this article, or claim that may be made by its manufacturer, is not guaranteed or endorsed by the publisher.

## Glossary

**Table:**

5-FU	5-fluorouracil
ABCB10	ATP binding cassette B family member 10
ADAM28	ADAM metallopeptidase domain 28
ADAM9	ADAM metallopeptidase domain 9
ADM	Adriamycin
AEG-1	Astrocyte-elevating protein-1
AK4	Adenylate kinase 4
AKT	Serine/threonine protein kinase B
ALKBH5	AlkB homolog H5
AML	Acute myeloid leukemia
AMPK	Activated protein kinase
ANLN	Anillin
ARNT2	Aryl hydrocarbon receptor nuclear translocator 2
ARv7	The androgen receptor splicing variant 7
ASAP1	Ankyrin repeat and Pleckstrin homology domain 1
ASK1	Apoptosis signal-regulating kinase 1
ATG2A	Autophagy-related gene 2A
ATG7	Autophagy Related 7
BCL2	B-cell lymphoma-2
BCL-2	B-cell lymphoma-2
BMI1	B cell-specific Moloney murine leukemia virus integration site 1
BRCA1	Breast cancer type 1 susceptibility protein
BTZ	Bortezomib
CAFs	Tumor-associated fibroblasts
CCDC66	Coiled-coil domain containing 66
CCNB1	Cyclin B1
CCNE1	Cyclin E1
CDCA3	Cell division cycle associated 3
CDDP	Cisplatin
CDK4	Cell cycle protein-dependent kinase 4
CDK6	Cyclin-dependent kinase 6
CEBPG	CCAAT enhancer binding protein gamma
CELSR1	Cadherin EGF LAG seven-pass G-type receptors 1
CHD4	Chromodomain helicase DNA-binding protein 4
Chk2	Checkpoint kinase 2
CHTOP	Chromatin target of protein arginine methyltransferase
circRNAs	Circular RNAs
CORO1C	Coronin 1C
CSC	Cancer stem cell
CSPP1	Centrosome and spindle pole associated protein 1
Cul2	Cullin2
CXCL10	Chemokine 10
CXCL12	Chemokine 12
CXCR4	Chemokine receptor type 4 DB
Diosbulbin-B	DCP1A
Decapping enzyme 1a	DDP
Diamminedichloroplatinum	DDP4
Dipeptidyl peptidase-4	DDR
DNA damage response	DDX17
DEAD-box helicase 17	DHX9
DExH-box helicase 9	DONSON
Downstream neighbor of SON	DOX
Doxorubicin	DPP4
Dipeptidyl peptidase 4	DTX
Docetaxel	DUSP7
Dual specificity phosphatase 7	E2F3
E2F transcription factor 3	ECM
Extracellular matrix	Eg5
Kinesin-5	EGFR
Epidermal growth factor receptor	EIF5A2
Eukaryotic translation initiation factor 5A-2	ELP3
Elongator complex protein 3	EMT
Epithelial-mesenchymal transition	Enz
Enzalutamide	ERK
Extracellular signal-regulated kinase	EZH2
Enhancer of zeste homolog 2	FAM73A
Family with sequence similarity 73	member A
FAT1	FAT atypical cadherin 1
FBXL18	F-box and leucine-rich repeat protein 18
FBXL5	Leucine-rich repeat protein 5
FBXW7	F-box and WD repeat domain containing 7
FGF9	Fibroblast growth factor 9
FGFR1	Fibroblast growth factor receptor 1
FMNL3	Formin like 3
FN1	Fibronectin 1
FOXC2	Forkhead box protein C2
FOXM1	Forkhead box protein M1
FOXO1	Forkhead box O1
FOXO3	Forkhead box O3
FOXO3a	Forkhead box O 3a
Foxp1	Forkhead box protein P1
FOXQ1	Forkhead box Q1
FOXR2	Forkhead box 2
FXR1	Fragile X-Related 1
FZD7	Frizzled-7
GEM	Gemcitabine
GFRA1	GDNF Family Receptor Alpha 1
GLUT1	Glucose transporter 1
GOT1	Glutamate oxaloacetate transaminase 1
GPCPD1	Glycerophosphocholine phosphodiesterase 1
GRB2	Growth factor receptor-bound protein-2
GRK5	G protein-coupled receptor kinase 5
H2AX	Histone family member X
HDGF	Hepatoma-derived growth factor
HECTD1	HECT domain E3 ubiquitin ligase 1
HGF	Hepatocyte growth factor
HIF-1α	Hypoxia-inducible factor-1α
HIPK2	Homeodomain-interacting protein kinase 2
HIPK3	Homeodomain-interacting protein kinase 3
HK2	Hexokinase-2
HMG	High mobility group
HMGA2	High-mobility group AT-hook 2
HMGB1	High-mobility group box 1
HNRNPK	Heterogeneous nuclear ribonucleoprotein K
HOXC8	Homeobox C8
IL-25	Interleukin -25
IM	Imatinib
ITCH	Itchy E3 ubiquitin protein ligase
JAG1	Jagged1
JAK2	Janus kinase 2
JMJD1C	Jumonji domain containing 1C
KANK1	Kidney ankyrin repeat-containing protein 1
KDM4C	Lysine demethylase 4C
KIF2A	Kinesin family member 2A
KLF12	Krüppel-like factor 12
LC3	Light chain 3
LDHA	Lactate dehydrogenase A
LIFR	Leukemia inhibitory factor receptor
LIN28B	Lin-28 homolog B
m6A	N6-methyladenosine
MCTP2	Multiple C2-domains with two transmembrane regions 2
MDM2	Mouse double minute 2
MDR1	Multidrug resistance gene 1
MDR-1	Multidrug resistance gene-1
MECP2	Methyl-CpG-binding-protein 2
MEK	Mitogen-activated protein kinase kinase
MET	MET proto-oncogene receptor tyrosine kinase
miRNAs	MicroRNAs
MMP1	Matrix metalloproteinase 1
MMP11	Matrix metalloproteinase 11
MMP17	Matrix metalloproteinase 17
MRP1	Multidrug resistance-associated protein-1
MSH2	MutS homolog 2
MSI1	Musashi1
MTA	Pemetrexed
MTMR3	Myotubularin-related protein 3
MTO1	Mitochondrial tRNA translation optimization 1
mTOR	Mechanistic target of rapamycin kinase
MTX	Methotrexate
MutSα	Alpha-melanocyte stimulating hormone
MYH9	Myosin heavy chain 9
nAChRα7	Nicotinic acetylcholine receptor alpha 7
NANOG	Nanog homeobox
NCOA3	Nuclear receptor coactivator 3
NEK2	NIMA-related kinase 2
NFIX	Nuclear factor I X
NK	Natural killer
NRAS	Neuroblastoma RAS
NRIP1	Nuclear receptor-interacting protein 1
NSCLC	Non-small-cell lung cancer
NT5E	Ecto-5’-nucleotidase
OXA	oxaliplatin
PBLD	Problem-based learning discussion
PBX3	Pre-leukemia transcription factor 3
PD-1	Programmed cell death 1
PDK1	3-phosphoinositide-dependent protein kinase 1
PD-L1	Programmed death-ligand 1
PDPK1	3-phosphatidylinositol-dependent protein kinase-1
PDX	Patient-derived xenograft
PGAM1	Phosphoglycerate mutase 1
PI3K	Phosphatidylinositol 3-kinase
PI3P	Phosphatidylinositol 3-phosphate
PIK3R1	The PI3K regulatory subunit p85alpha
PIP5K1A	Phosphatidylinositol-4-phosphate 5-kinase type 1 alpha
PKM2	The M2 isoform of pyruvate kinase
PKP3	Plakophilin 3
POLD4	Eukaryote DNA polymeraseδ4
POU3F2	POU class 3 homeobox 2
PPFIA1	PTPRF interacting protein alpha 1
PRDM2	PR/SET domain 2
PRDX2	Peroxiredoxin 2
PRKCD	Protein kinase C, delta
PRPF39	Pre-mRNA splicing factor 39
PSMC3	Proteasome 26S ATPase subunit 3
PTEN	Phosphatase and tensin homolog
PTGR1	Effect of prostaglandin reductase 1
PTX	Paclitaxel
PVT1	Plasmacytoma variant translocation 1
RAS	Rat sarcoma
RASSF1	RAS-Association Domain Family 1
RASSF6	Ras association domain family member 6
RB	Retinoblastoma protein
RBP	RNA binding protein
REV3L	EV3-like DNA-directed polymerase ζ catalytic subunit
RNF	RING finger protein
ROCK	Rho-associated coiled-coil-forming protein kinase
ROR1	Receptor tyrosine kinase-like orphan receptor 1
S100A1	S100A1 calcium-binding protein A1
SAE	SUMO-activating enzyme subunit
SAE2	Activating enzyme subunit 2
SCAI	Suppressor of Cancer Cell Invasion
SCMH1	Sex comb on midleg homolog-1
SEMA6D	Semaphorins 6D
SH2	Src homology region 2
SHP2	SH2-containing protein tyrosine phosphatase 2
SIK2	Salt inducible kinase 2
SKA1	Spindle and kinetochore-associated complex subunit 1
SNAI1	Snai family zinc finger 1
SNX6	Sorting nexin 6
SOX4	SRY-box transcription factor 4
SOX9	SRY-box transcription factor 9
STAT3	Signal transducer and activator of transcription 3
STX3	Syntaxin 3
SUMO	Small ubiquitin-related modifier
TAM	Tamoxifen
TAMs	Tumor-associated macrophages
TGF-α	Transforming growth factor alpha
TGM2	Transglutaminase-2
TIM-3	T cell immunoglobulin and mucin domain 3
TKI	Tyrosine kinase inhibitor
TKIs	Tyrosine kinase inhibitors
TLR4	Toll-like Receptor 4
TME	Tumor microenvironment
TMZ	Temozolomide
TNPO3	Transportin3
TPD52	Tumor protein D52
TRAF4	Tumor necrosis factor receptor associated factor 4
TRIM14	Tripartite motif-containing 14
TRIM65	Tripartite motif-containing protein 65
TWIST1	Twist family bHLH transcription factor 1
UBAP2	Ubiquitin associated protein 2
UHRF1	Ubiquitin like with PHD and ring finger domains 1
ULK1	Unc-51-like kinase 1
USP7	Ubiquitin-specific protease-7
VapA	Virulence associated protein A
VEGFA	Vascular endothelial growth factor A
VRACs	Volume-regulated anion channels
WIF1	Wnt inhibitory factor 1
Wnt7a	Wnt family member 7A
WTAP	Wilms tumor 1-associated protein
XIAP	X-linked inhibitor of apoptosis protein
YAP	Yes-associated protein
YAP1	Yes-associated protein 1
YBX1	Y box binding protein 1
ZEB1	Zinc finger E-box binding homeobox 1
ZIC5	Zinc finger protein of the cerebellum 5
